# A protein of the metallo-hydrolase/oxidoreductase superfamily with both beta-lactamase and ribonuclease activity is linked with translation in giant viruses

**DOI:** 10.1038/s41598-020-78658-8

**Published:** 2020-12-10

**Authors:** Philippe Colson, Lucile Pinault, Said Azza, Nicholas Armstrong, Eric Chabriere, Bernard La Scola, Pierre Pontarotti, Didier Raoult

**Affiliations:** 1 Aix-Marseille Univ., Institut de Recherche pour le Développement (IRD), Assistance Publique - Hôpitaux de Marseille (AP-HM), Microbes Evolution Phylogeny and Infections (MEPHI), 27 boulevard Jean Moulin, 13005 Marseille, France; 2 IHU Méditerranée Infection, 19-21 boulevard Jean Moulin, 13005 Marseille, France; 3 CNRS, Marseille, France

**Keywords:** Microbiology, Diseases

## Abstract

Proteins with a metallo-beta-lactamase (MBL) fold have been largely studied in bacteria in the framework of resistance to beta-lactams, but their spectrum of activities is broader. We show here that the giant Tupanvirus also encodes a MBL fold-protein that has orthologs in other giant viruses, a deep phylogenetic root and is clustered with tRNases. This protein is significantly associated with translation components in giant viruses. After expression in *Escherichia coli*, it was found to hydrolyse nitrocefin, a beta-lactam, and penicillin G. This was inhibited by sulbactam, a beta-lactamase inhibitor. In addition, the tupanvirus MBL fold-protein was not active on single- or double-stranded DNA, but degraded RNAs from bacteria and *Acanthamoeba castellanii*, the tupanvirus amoebal host. This activity was not neutralized by sulbactam. Overall, our results still broaden the host range of MBL fold-proteins, showing dual beta-lactamase/nuclease activities in giant viruses.

## Introduction

The metallo-hydrolase/oxidoreductase superfamily encompasses a large set of enzymes, including metallo-beta-lactamase (MBL) and ribonuclease (RNase) Z enzymes^1,2^. These enzymes are pleiotropic proteins that have native and promiscuous activities and can hydrolyze a wide range of substrates, like beta-lactams, and DNA or RNA^3–5^. Such capabilities rely on an ancient and highly conserved fold, which represents a stable scaffold that has evolved to perform a broad range of chemical reactions and on which various catalytic, regulatory and structural activities are based^3,4,6^. This wide array of activities is enabled by variations in the composition and size of loops located near the enzyme active site^4^. A well-known catalytic activity of MBLs particularly studied in clinical microbiology consists in breaking beta-lactam rings, which was primarily identified in bacteria^7^. Nevertheless, this hydrolase activity is suspected to have evolved in response to the environmental beta-lactams from an ancestral protein whose function was not related to beta-lactams and which may have been devoid of such hydrolase capability^4^. Concurrently to their capability to interact with various substrates that likely emerged through adaptive evolution, members of the metallo-hydrolase/oxidoreductase superfamily have been identified in a broad range of cellular organisms, including bacteria, but also eukaryotes and archaea with a beta-lactamase activity^3,8^.

Giant viruses are *bona fide* microbes as their virions are visible under a light microscope and they display a complexity similar to that of small cellular microorganisms^9,10^. Since their discovery in 2003, their diversity has increased considerably, with nine families and more than 100 isolates cultured. Their classification alongside cellular microorganisms is still debated, but their characteristics clearly distinguish them from conventional viruses^11,12^. We have investigated whether genes encoding members of the metallo-hydrolase/oxidoreductase superfamily may also be present in giant viruses. We found one in Tupanvirus deep ocean, a giant mimivirus isolated from Brazilian Atlantic ocean sediments, and confirmed that its product harbored a biologically active MBL fold with both RNase and beta-lactamase activities, which may be native and promiscuous activities, respectively.

## Results

**Figure 1:**
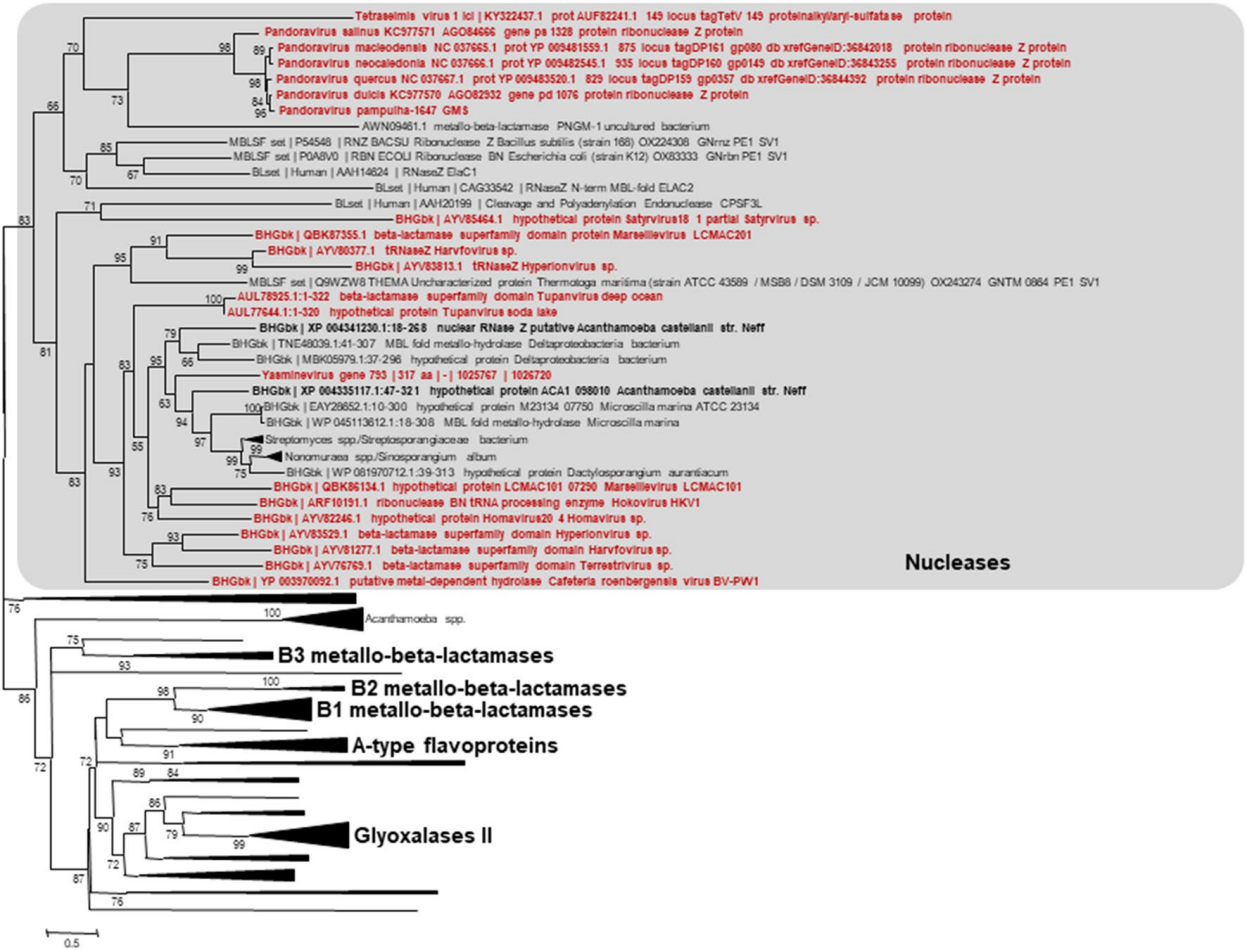
Phylogeny reconstruction based on metallo-beta-lactamase (MBL) fold proteins. Phylogeny reconstruction was performed after amino acid sequence alignment with the Muscle program^34^ with the Maximum-Likelihood method using FastTree^35^, and tree was visualized with the MEGA 6 software^36^. The amino acid sequences analyzed are Tupanvirus deep ocean protein AUL78925.1 and its homologs with the greatest BLASTp scores from the NCBI GenBank protein sequence database (nr) (see Supplementary Table 1), our sequence database of giant virus genomes, and previously described draft genome sequences from 14 *Acanthamoeba* species^37^; a set of previously described MBL fold proteins^21^; and a set of sequences from the UniProtKB database^1^, previously used for phylogeny reconstructions. Extended tree is available in Supplementary Fig. 1.

While annotating the genome of Tupanvirus deep ocean, the second isolate of a new mimivirus genus, *Tupanvirus*^13^, a gene (GenBank: AUL78925.1) that encodes a metallo-hydrolase-like MBL fold was identified (Conserved Protein Domain Family Accession no. cl23716)^14^. This gene has a homolog in the other tupanvirus isolate (Soda Lake) (AUL77644.1). Beyond, best BLASTp hits against cellular organisms included MBL fold harboring proteins from an unclassified deltaproteobacterium whose genome was assembled from a marine water metagenome (e-value, 5e−38; identity, 33.0%; coverage, 83%), from an actinobacteria (*Nonnomuraea* spp.) (1e−36; 30.0; 86%), from *Microscilla marina* (6e−34; 28.5%; 89%) and from *Acanthamoeba castellanii* (4e−33; 29.8%; 81%) (Fig. 1; see Supplementary Fig. 1). Significant BLASTp hits (e-values ranging from 1e−41 to 8e−6) against viruses were also obtained with genes from putative giant viruses whose genomes were assembled from metagenomes obtained from environmental samples^15–17^ and from Cafeteria roenbergensis virus, a distant Mimivirus relative^18^. The 322 amino acid long tupanvirus protein exhibits the conserved MBL motif "HxHxDH" in amino acid positions 60–65 (H60-H62-H65). Besides, two putative metal-binding sites, H60-H62-H154 and D64-H65-H268, may be underscored. A search for domains using the NCBI conserved domain search (CD Search) tool^19^ identified a MBL fold belonging to a ribonuclease Z (RNase_Z_T_toga, TIGR02650, interval = amino acids 24-273, E-value = 1.81e−14; RNaseZ_ZiPD-like_MBL-fold, cd07717, interval = amino acids 56-282, E-value = 1.63e−04), which is a transfer RNA (tRNA)-processing endonuclease. This Tupanvirus deep ocean protein was further analyzed by three-dimensional comparison against the Phyre2 web portal for protein modeling, prediction and analysis^20^. This analysis reported a best match with 100% confidence and 85% coverage (273 amino acid residues) with the crystal structure of a long form ribonuclease Z (RNase Z) from yeast (template c5mtzA) (see Supplementary Fig. 2, Supplementary File 1). Proteome analysis conducted as previously described^14^ on Tupanvirus Soda Lake and Tupanvirus deep ocean virions did not detect proteins with a MBL fold. In addition, transcriptomic analyses could not be performed due to the dramatic RNA shutdown observed during the tupanvirus replication^14^. Interestingly, the genomes of 20 of the 21 (95%) giant viruses found to encode a MBL fold protein concurrently encode tRNAs. This is only the case for 46 of the 122 (38%) giant virus genomes not encoding a MBL fold protein (*p* < 10^–3^; Yates-corrected chi-square test) (see Supplementary Figs. 3 and 4 and Supplementary Table 2). The presence of a MBL fold protein among Megavirales members was correlated with the size of the gene repertoire and the number of translation-associated components (*p* < 10^–3^; Anova test). Putative proteins with a MBL fold from giant viruses comprised two related phylogenetic clusters (Fig. 1). These clusters appeared deeply rooted in the phylogenetic tree, which suggests an ancient origin for these genes. In addition, one of the clusters of giant virus genes encoding MBL fold proteins appeared closely related to two genes from *Acanthamoeba castellanii*, an amoebal mimivirus hosts, suggesting a transfer from these giant viruses to *A. castellanii*.

**Figure 2:**
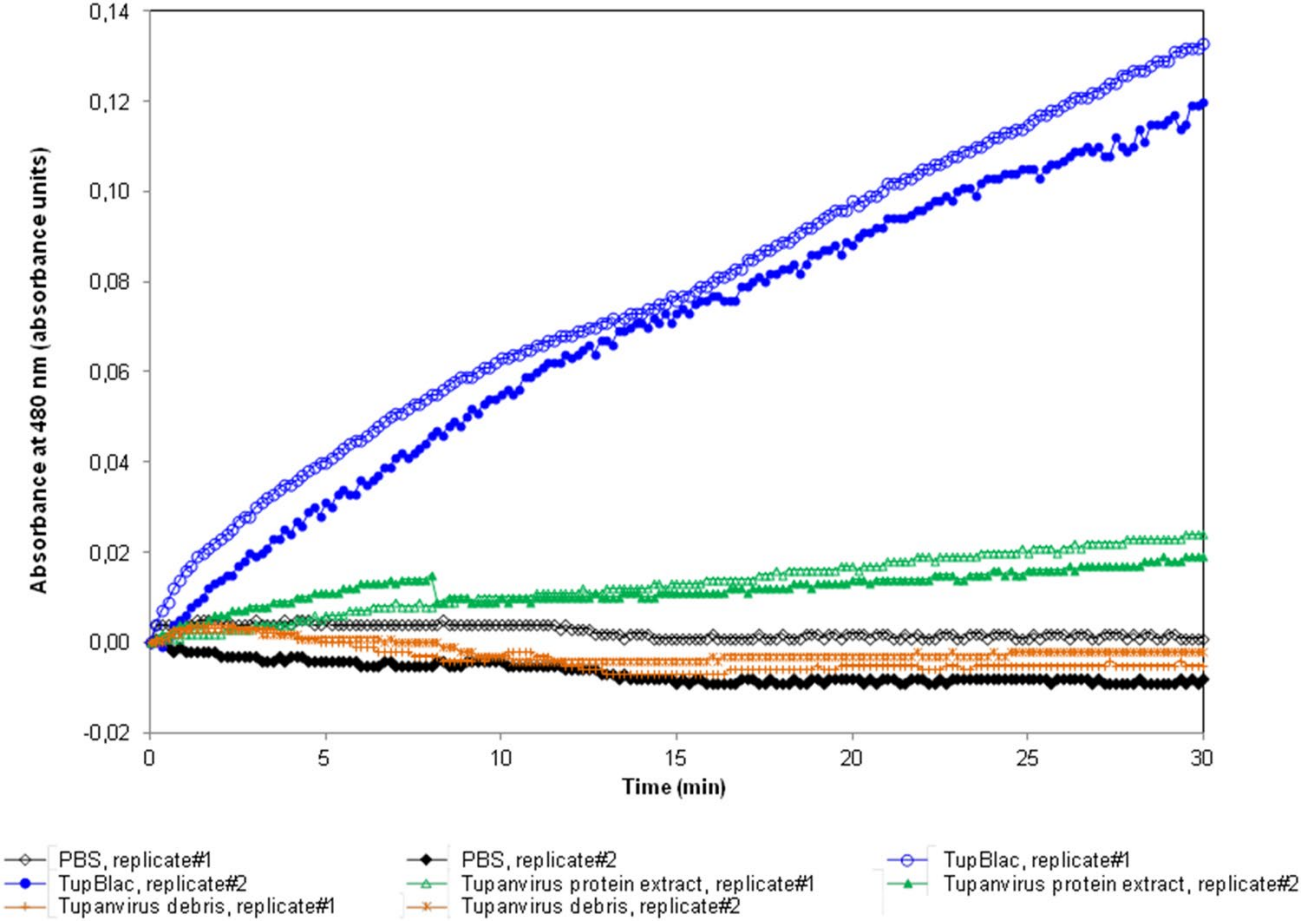
Effect on nitrocefin of expressed Tupanvirus protein (TupBlac). The effect on nitrocefin of the expressed Tupanvirus protein extract (TupBlac) was assessed by monitoring the degradation of nitrocefin, a chromogenic cephalosporin substrate. Tupanvirus protein extract and tupanvirus debris were concurrently tested. PBS, Phosphate-Buffered Saline; TupBlac, tupanvirus expressed protein.

**Figure 3:**
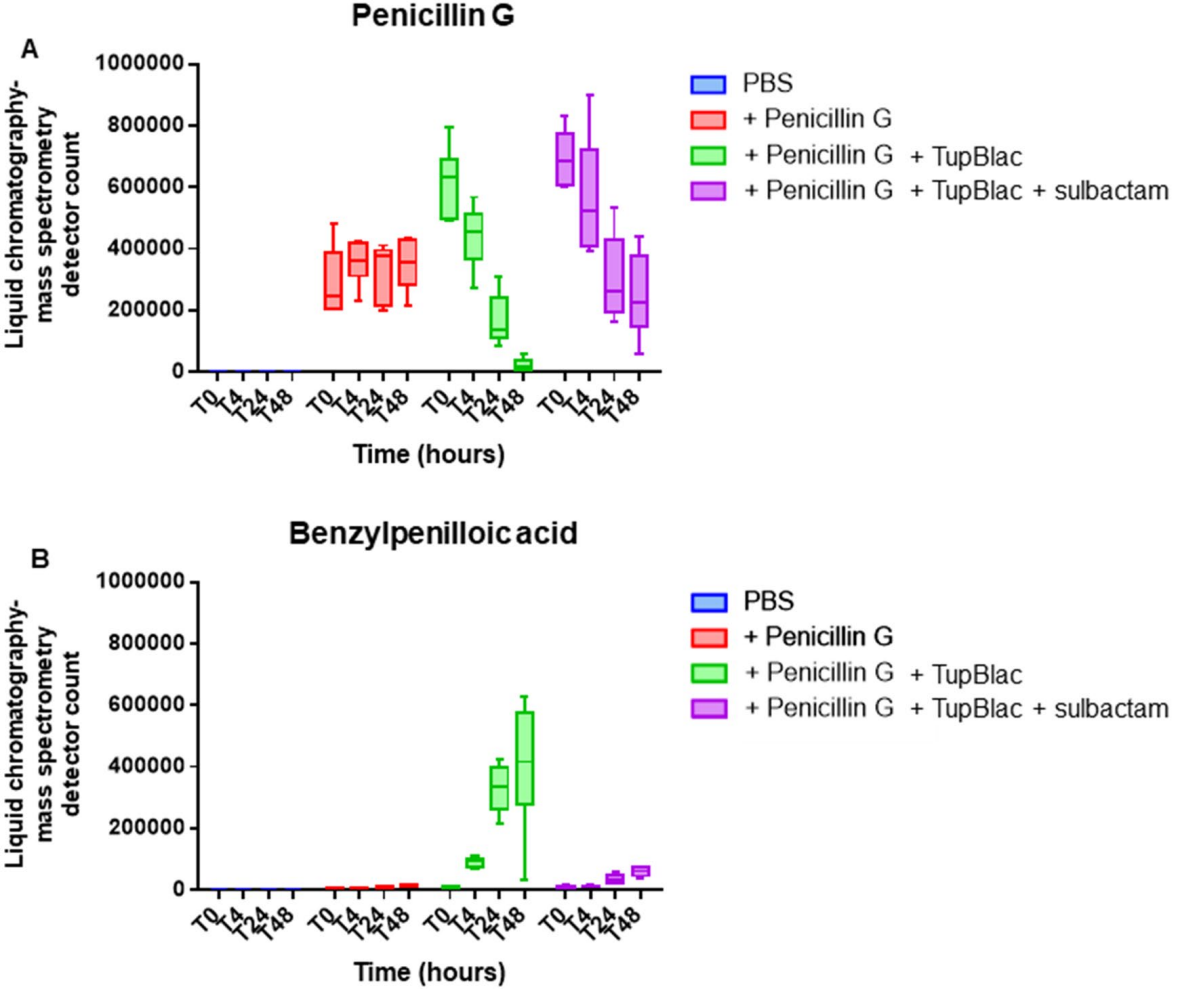
Effect on penicillin G of expressed Tupanvirus protein (TupBlac). The effect on penicillin G of the expressed Tupanvirus protein (TupBlac) and its inhibition by sulbactam were assessed by monitoring by liquid chromatography-mass spectrometry (LC-MS) the degradation of penicillin G (**A**) and the appearance of benzylpenilloic acid, the metabolite resulting from the enzymatic hydrolysis of penicillin G (**B**), at times (T) T0 (0 h), T4 (4 h), T24 (24 h), and T48 (48 h). PBS, Phosphate-Buffered Saline; TupBlac, tupanvirus expressed protein.

**Figure 4:**
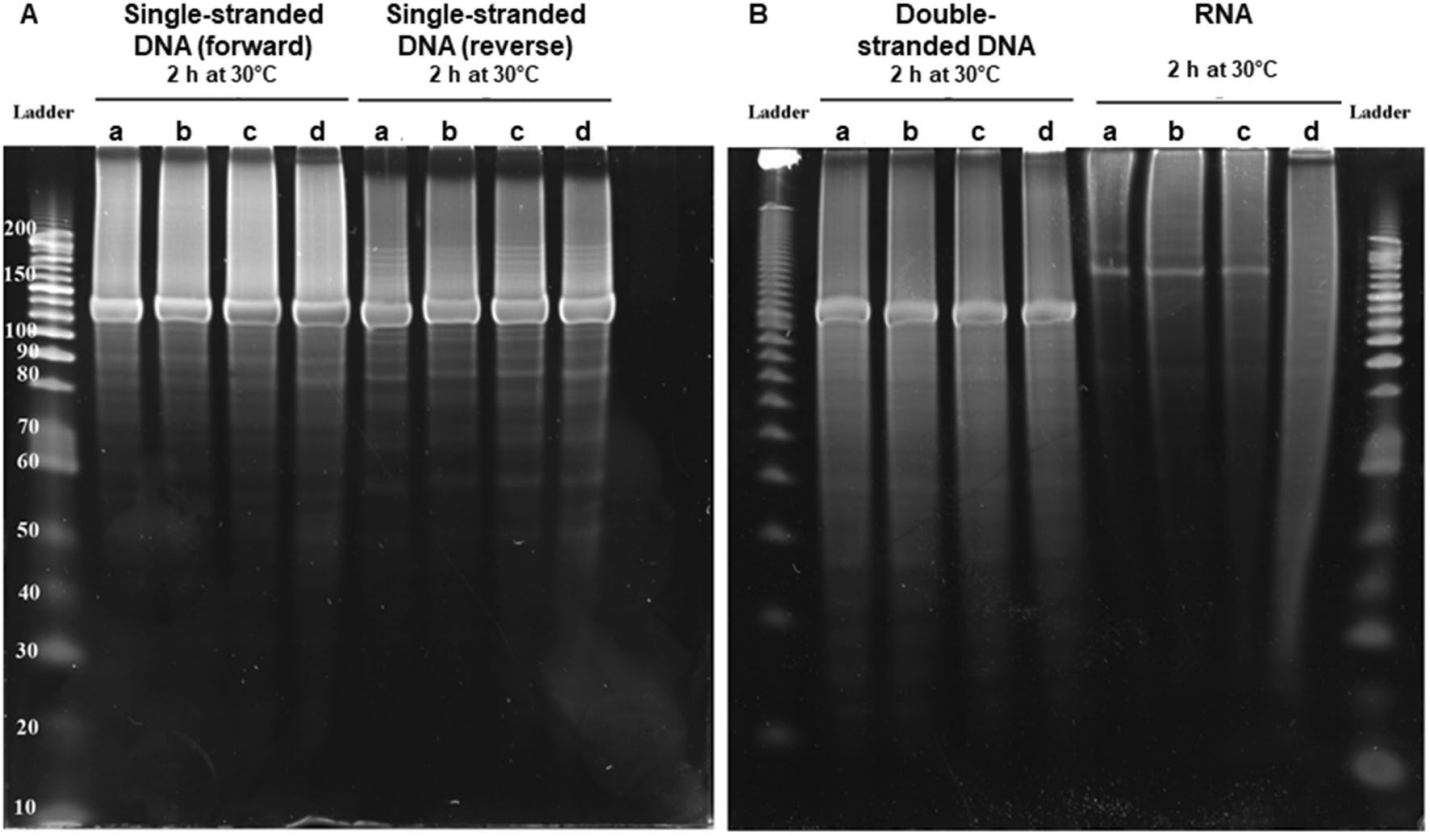
Nuclease activity on various types of nucleic acids of expressed Tupanvirus protein (TupBlac) as assessed by dPAGE. Denaturant polyacrylamide gel electrophoresis (12% dPAGE) of nuclease activity on synthetic (+) and (−) single-stranded DNAs (130 nucleotide-long) (**A**), synthetic double-stranded DNA (**B**) (see Supplementary Table 3), or *Escherichia coli* RNA (**B**). Each part A and B of the figure correspond to different gels. No treatment (a); buffer (b); succinate dehydrogenase enzyme produced and purified by the same process and collected in the same fractions as Tupanvirus beta-lactamase TupBlac, used as negative control (c); Tupanvirus beta-lactamase TupBlac (d).

The recombinant protein AUL78925.1 of Tupanvirus deep ocean (named TupBlac) was expressed in *Escherichia coli* and was then purified, as described previously^8^. Based on the phylogenetic analysis and as MBL folds can hydrolyse nucleic acids^3^, both beta-lactamase and nuclease activities of this purified protein were thereafter tested. We first evaluated the beta-lactamase activity of a pure solution of TupBlac used at a concentration of 1 µg/mL by incubating it with nitrocefin, a chromogenic beta-lactam used to test the beta-lactamase activity^21^. A significant hydrolysis activity was observed (Fig. 2). A concentrate of protein extract (50 mg/mL) obtained from tupanvirus virions also degraded, albeit slightly, nitrocefin. According to Michaelis–Menten equation fitting (R^2^ = 0.97), the following kinetic parameters for TupBlac against nitrocefin were estimated: kcat = 8.8 × 10^–4^ ± 8.5 × 10^–5^ s^−1^, Km = 160 ± 5 µM and kcat/Km = 5.5 s^−1^ M^−1^ (see Supplementary Fig. 5). They were indicative of a promiscuous activity^2^. Thereafter, we monitored by liquid chromatography-mass spectrometry the effect of TupBlac on penicillin G (10 µg/mL) and observed a significant hydrolysis activity of this coumpound within 48 h (Fig. 3). We also detected, in the presence of the tupanvirus protein, benzylpenilloic acid, the metabolite resulting from the enzymatic hydrolysis of penicillin G^22^. Finally, we confirmed that these observations were related to a beta-lactamase activity as both penicillin G degradation and benzylpenilloic acid appearance were inhibited by sulbactam, a beta-lactamase inhibitor (Fig. 3). We further tested if pre-treatment with sulbactam had an impact on the duration of the giant virus replication cycle and replication intensity. After replication on *A. castellanii* strain Neff in the presence of a high concentration (10 µg/mL) of sulbactam, the virions produced (10^6^/mL) were inoculated on fresh amoebae at different concentrations. No differences were observed regarding viral growth in the absence or presence of pre-treatment with sulbactam as assessed using high content screening (see Supplementary Fig. 6).

**Figure 5:**
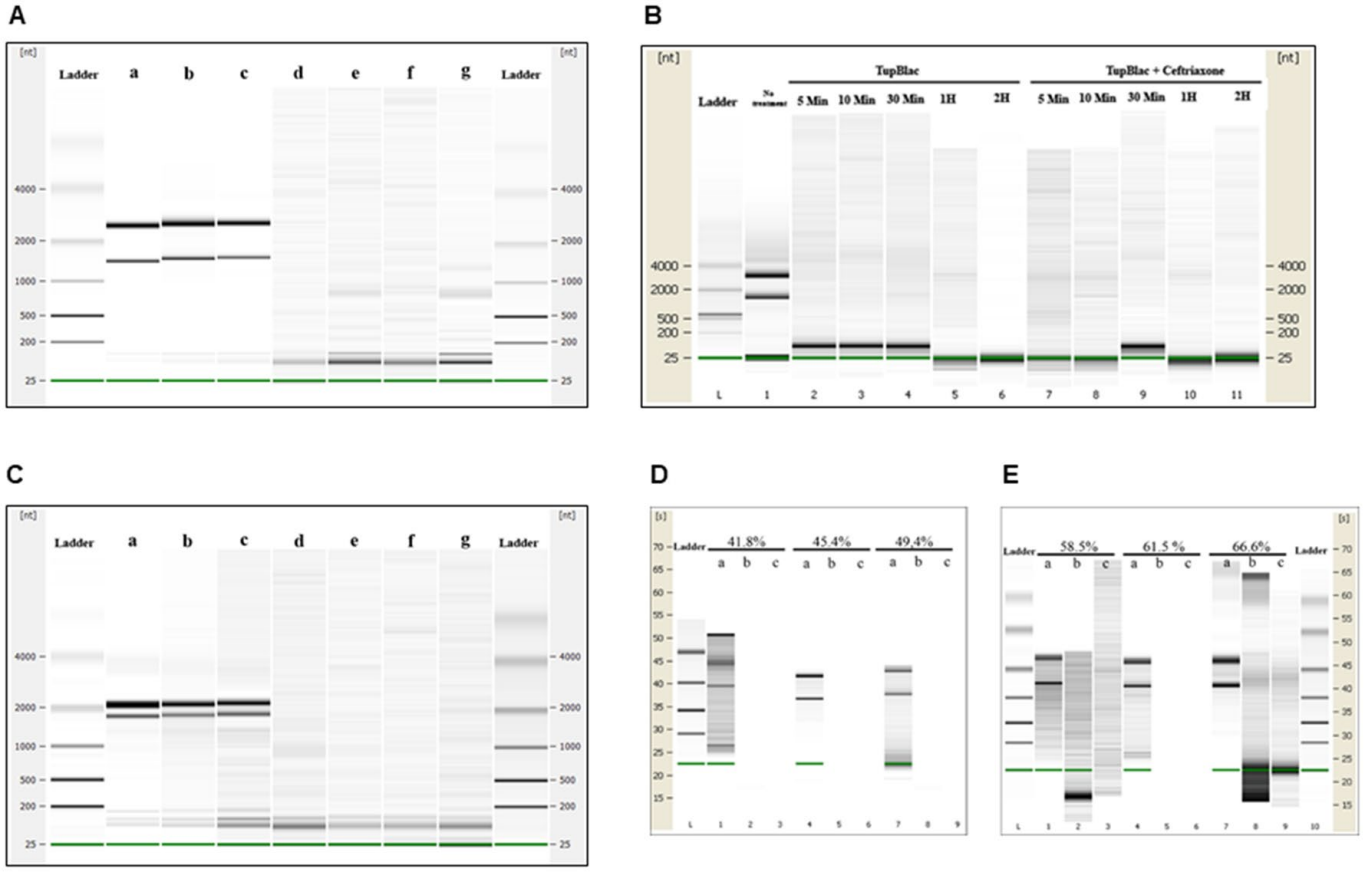
Digital gel images of RNase activity of expressed Tupanvirus protein TupBlac on RNAs originating from *E. coli, Acanthamoeba castellanii*, and bacteria that differ by the G + C-content of their genome. RNA samples (1 µg) were incubated with 15 µg of TupBlac at 30 °C in the absence or presence of 10 µg/mL of sulbactam or 200 µM of ceftriaxone. Nuclease activity was visualized as digital gel images performed using the Agilent Bioanalyzer 2100 with the RNA 6000 Pico LabChip (Agilent Technologies, Palo Alto, CA). Each part A to E of the figure correspond to different digital gel images generated by the Agilent Bioanalyzer. (**A**) RNA used as substrate was from *Escherichia coli;* no treatment (a); buffer (b); blank control made with bacterial lysates purified by the same process and collected as Tupanvirus beta-lactamase (c); TupBlac in the absence (d) or presence (e) of 10 µg/mL of sulbactam, of 200 µM of ceftriaxone (f), or of 10 mM of EDTA (g). (**B**) RNA used as substrate was from *E. coli;* reactions were stopped at different times (5 min, 10 min, 30 min, 1 h and 2 h) by the addition of proteinase K (10 µg) and incubation for 1 h at 37 °C. The first lane corresponds to no treatment; lanes 2 to 6 correspond to RNA treatment with TupBlac in the absence of ceftriaxone; lanes 7 to 11 correspond to RNA treatment with TupBlac in the presence of ceftriaxone. (**C**) Nuclease activity on RNAs originating from *Acanthamoeba castellanii*; no treatment (a); buffer (b); blank control (c); TupBlac in the absence (d) or presence (e) of sulbactam (10 µg/mL), ceftriaxone (200 µM) (f), or EDTA (10 mM) (g). (**D**,**E**) nuclease activity on RNAs originating from bacteria that differ by the G + C-content of their genome, as indicated at the top of the digital gel image. For each RNA, three samples were analyzed: no treatment (a); treatment with TupBlac in the absence of sulbactam (b); and treatment with TupBlac in the presence of sulbactam (c).

Finally, as some proteins with a MBL fold can hydrolyse DNA and RNA^3^, we tested the capability of tupanvirus enzyme TupBLac to degrade synthetic single- and double-stranded DNAs and bacterial RNAs. We found no effect on both DNA types. In contrast, we observed a strong RNase activity (Fig. 4). Another set of experiments was conducted using *E. coli* RNA as a substrate with an assessment of RNA size distribution on a bioanalyzer (Agilent Technologies, Palo Alto, CA) after incubation with TupBlac. It showed a dramatic degradation of RNAs by the tupanvirus enzyme (Fig. 5A). In contrast with the beta-lactamase activity, this was not inhibited, neither by sulbactam (Fig. 5A, and see Supplementary Fig. 7), nor by ceftriaxone (Fig. 5B), a cephalosporin that inhibits human SNM1A and SNM1B, that are DNA repair nucleases with a MBL fold^23^. This RNase activity was not inhibited by EDTA either. In addition, a RNase activity of the Tupanvirus protein was further observed on *A. castellanii* RNA, and not inhibited either by sulbactam, ceftriaxone or EDTA (Fig. 5C). TupBLac also degraded RNA extracted from bacteria with genomes with different G + C contents ranging between 41.8% and 66.6% (Fig. 5D,E), suggesting an absence of influence of the G + C richness on the RNase activity. Finally, TupBlac RNase activity was estimated to be 0.451 ± 0.153 mU/mg using a fluorescence-based assay, without difference in the presence of sulbactam or ceftriaxone (0.520 ± 0.003 and 0.551 ± 0.024 mU/mg, respectively) (see Supplementary Fig. 8).

## Discussion

Hence, we found herein by several bioinformatic approaches that a gene of Tupanvirus deep ocean, a recently discovered giant virus classified in family *Mimiviridae*^13,14^, encodes a protein with a MBL fold. We further observed that this protein exhibited dual beta-lactamase and RNase activities. This is the first evidence of the presence of a biologically-active protein with a MBL fold in a virus. In addition, this work parallels the one on a protein detected by functional screening of a metagenomic library from the deep-seep sediments^24^, showing that the same enzyme has both beta-lactamase and RNase activities. It is noteworthy that the beta-lactamase activity of the MBL fold protein of Tupanvirus was inhibited by a beta-lactamase inhibitor but this was not the case for the RNase activity^2^. It is also worthy to consider that this tupanvirus protein with a MBL fold may display other enzymatic activities that were not tested here, as such proteins are known to be pleiotropic^4^. As a matter of fact, the existence of promiscuous activities in some proteins indicates that these latter can evolve to perform a broad range of functions, according to the environmental settings^2,4,5^. Thus, the MBL fold can be used to perform various enzymatic activities.

The phylogenetic study of this MBL fold protein shows the presence in several other giant viruses of phylogenetically-clustered counterparts, the origin of which seems very old. Interestingly, it also appears that there may have been a gene transfer between these giant viruses and *Acanthamoeba* sp., the amoebal host of many giant viruses. Such potential for horizontal transfer of these MBL fold proteins is well-recognized^4^. Beta-lactamases are a priori useless for giant viruses, which are grown in the presence of various antibiotics, including beta-lactams^25^. The recent description of penicillin secretion by arthropods^26^ and the demonstration of active enzymes belonging to the metallo-hydrolase/oxidoreductase superfamily in vertebrates including humans^21^, as well as in archaea^8^, fungi^27^ and now viruses show that MBL fold proteins have a dramatically broad distribution.

In humans, 18 genes were annotated as beta-lactamases, whose activity had not been biologically-tested until recently^21^. In addition, MBL fold proteins were highlighted to digest DNA or RNA^3,21^. Thus, a class of enzymes, that were named beta-lactamases because of their original discovery in bacteria resistant to beta-lactamines, are in fact potentially versatile proteins. This differs from the drastically-simplified paradigm consisting in enzymes with a beta-lactamase activity being secreted by bacteria under the selective pressure of natural or prescribed antibiotics.

The RNase activity observed here for the Tupanvirus MBL fold protein could be related to the host ribosomal shutdown observed in the presence of Tupanvirus deep ocean with various protists, the mechanism of which has not been elucidated^14^. This activity could allow these viruses to take over on their cellular hosts by degrading cellular messenger RNAs and shutting down cellular gene expression. The giant virus mRNAs should be protected from such a degradation, which may be explained by the encapsidation of RNA transcripts into giant virions that was detected for some of these viruses^28^. Bioinformatic analyses suggested that the tupanvirus MBL fold protein may belong to the RNase Z group that was proposed to be one of the two main groups of the metallo-hydrolase/oxidoreductase superfamily encompassing MBLs^1^. RNase Z enzymes perform tRNA maturation by catalyzing the endoribonucleolytic removal of the 3’ extension of tRNA precursors that do not contain a chromosomally-encoded CCA determinant^29–31^. The presence in giant viruses of RNases showing the greatest homology to tRNases suggests a specific activity on tRNAs, which seems consistent with the presence of a large set of translation components in these viruses, first and foremost Tupanvirus deep ocean that is the current record holder of the number of translation components (including 70 tRNAs targeting all 20 canonical amino acids). The presence of a putative tRNase in the virus that currently has the most complete set of translation components of the whole virosphere is likely not fortuitous. Furthermore, it was described for *Escherichia coli* that its RNase Z had endoribonucleasic activity on messager RNAs, being responsible for their decay in in vitro experiments^30^. This further argues that MBL fold proteins may contain a wide range of activities^5^. PNGM-1, a MBL fold protein whose sequence was recently described from a deep-sea sediment metagenome by detection of its beta-lactamase activity^32^, was also found to harbor dual beta-lactamase and RNase activities^24^. MBL fold proteins from giant viruses are clustered with this protein in the phylogenetic analysis. Interestingly, PNGM-1 was suspected to have evolved from a tRNase Z^24^. In conclusion, our data still broaden the range of biological hosts of MBL fold proteins and further demonstrate that such proteins display broad enzymatic activity.

## Materials and methods

### Bioinformatics

Searches for Tupanvirus deep ocean protein AUL78925.1 homologs were performed using the BLAST tool^33^. Phylogeny reconstruction was performed after amino acid sequence alignment with the Muscle program^34^ and the Maximum-Likelihood method using FastTree^35^, and tree visualization used MEGA 6 software^36^. The amino acid sequences analyzed are Tupanvirus deep ocean protein AUL78925.1 and its homologs with the greatest BLASTp scores from the NCBI GenBank protein sequence database, our sequence database of giant virus genomes, and previously described draft genome sequences from 14 *Acanthamoeba* species^37^; a set of previously described MBL fold proteins^21^; and a set of sequences from the UniProtKB database^1^ previously used for phylogeny reconstructions. Three-dimensional comparisons for protein modeling, prediction and analysis were carried out against the Phyre2 web portal^20^. The set of translation components from each representative of the proposed order Megavirales^38^ was obtained through a BLASTp search^33^ with their repertoire of predicted proteins against clusters of orthologous groups of proteins (COGs) involved in translation (category J)^39^, using 10^–4^ and 50 amino acids as thresholds for e-values and sequence alignment lengths, respectively. The set of tRNAs from each virus was collected using the ARAGORN online tool (http://130.235.244.92/ARAGORN/)^40^. Hierarchical clustering was performed using the MultiExperiment Viewer software^41^ based on the patterns of presence/absence of MBL fold protein, numbers of translation-associated components (number of tRNAs, aminoacyl tRNA-synthetases, other tRNA-associated proteins, other translation-associated proteins) and size of the gene repertoires for Megavirales members (see Supplementary Table 2). For each item, the maximum value was determined, and values for each virus were considered relatively to these maximum values, being therefore comprised between 0 and 100%.

### Cloning, expression and purification

The Tupanvirus deep ocean gene bioinformatically predicted to encode a beta-lactamase superfamily domain (AUL78925.1^14^) was designed to include a Strep-tag at the N-terminus and optimized for its expression by *Escherichia coli*. It was synthetized by GenScript (Piscataway, NJ, USA) and ligated between the NdeI and NotI restriction sites of a pET24a(+) plasmid. *E. coli* BL21(DE3)-pGro7/GroEL (Takara Shuzo Co., Kyoto, Japan) grown in ZYP-5052 media were used for the expression of the recombinant protein, under double antibiotic selection including with chloramphenicol and kanamycin. When the culture reached an O.D._600 nm_ = 0.6 at 37 °C, the temperature was lowered to 20 °C and L-arabinose (0.2% m/v) was added in order to induce the expression of chaperones. After 20 h, cells were harvested by centrifugation (5,000 g, 30 min, 4 °C) and the pellet was resuspended in washing buffer (50 mM Tris pH 8, 300 mM NaCl) and then stored at −80 °C overnight. Frozen *E. coli* were thawed and incubated on ice for 1 h after having added lysozyme, DNAse I and PMSF (phenylmethylsulfonyl fluoride) to final concentrations of 0.25 mg/mL, 10 µg/mL and 0.1 mM, respectively. Partially lysed cells were then disrupted by 3 consecutive cycles of sonication (30 s, amplitude 45) performed on a Q700 sonicator system (QSonica). Cellular debris were discarded following a centrifugation step (10,000 g, 20 min, 4 °C). The Tupanvirus protein was purified with an ÄKTA avant system (GE Healthcare, Bucks, UK) using Strep-tag affinity chromatography (wash buffer: 50 mM Tris pH 8, 300 mM NaCl, and elution buffer: 50 mM Tris pH 8, 300 mM NaCl, 2.5 mM desthiobiotin) on a 5 mL StrepTrap HP column (GE Healthcare). Fractions containing the protein of interest were pooled. Protein purity was assessed using 12.5% SDS-PAGE analysis (Coomassie staining). Protein expression was confirmed by performing MALDI-TOF MS analysis on gel bands previously obtained by SDS-PAGE. Protein concentrations were measured using a Nanodrop 2000c spectrophotometer (Thermo Scientific, Madison, WI, USA).

### Spectrophotometry assay for the detection of beta-lactamase activity in Tupanvirus virions

Tupanvirus purified virions in solution were centrifuged at 5,000 RPM in order to collect 1 g of humid matter. Virions were then suspended into 2 mL of a phosphate-buffered saline (PBS) solution at pH 7.4 prepared in water from a commercial salt mixture (bioMerieux, Marcy-l'Etoile, France). Virions were broken after five freeze–thaw cycles followed by 10 min of sonication (Q700 sonicator with a Cup Horn, QSonica, Newtown, Connecticut, USA). Integrity of virions was checked by scanning electron microscopy (TM 4000, Hitachi High-Technologie Corporation, Tokyo, Japan). Debris were discarded following a centrifugation step (15,000 *g*, 10 min). The clear supernatant was lyophilized and then reconstituted in 100 μL of PBS (corresponding to a final concentration of 50 mg/mL of soluble proteins). A pure solution of Tupanvirus protein was buffer-exchanged in PBS and the concentration was adjusted to 1 mg/ml. The degradation of nitrocefin (1 mM in PBS), a chromogenic cephalosporin substrate, was monitored as previously described after the addition of virion protein extract or Tupanvirus protein to the solution^7^. In order to determine kinetic parameters, initial velocities were calculated by Gen5.1 software (BioTek, Winooski, VT, USA) and obtained mean values were fitted using the Michaelis–Menten equation on Prism 6 (GraphPad Software, San Diego, CA, USA).

### Beta-lactam antibiotic degradation monitoring by liquid chromatography-mass spectrometry (LC-MS)

Penicillin G and sulbactam stock solutions at 10 mg/mL were freshly prepared in water from the corresponding high purity salts (Sigma Aldrich). A total of 30 μL of tupanvirus protein solution at 1 mg/mL was spiked with penicillin G and sulbactam at a final concentration of 10 μg/mL, before incubation at room temperature. Each time point corresponded to triplicate sample preparations. Negative controls consisted of PBS spiked with penicillin G and sulbactam. Then, 70 μL of acetonitrile were added to each sample, and tubes were vortexed 10 min at 16,000 *g* to precipitate the proteins. The clear supernatant was collected for analysis using an Acquity I-Class UPLC chromatography system connected to a Vion IMS Qtof ion mobility-quadrupole-time of flight mass spectrometer, as previously described^8^.

### Assessment of the effect of a beta-lactamase inhibitor on Tupanvirus growth

To evaluate the effect of a beta-lactamase inhibitor sulbactam on Tupanvirus growth, we tested Tupanvirus replication on *A. castellanii* pre-incubated with a high dose of sulbactam. Tests were performed in triplicate and amoebae cultivated in trypticase soy medium^14^. Four 1 mL culture wells containing 5.10^5^ *A. castellanii* were incubated at 32 °C, one of which contained 500 mg/L of sulbactam. After 24 h, Tupanvirus was added at a multiplicity of infection (MOI) of 1 in the well with sulbactam. Two other wells were inoculated with Tupanvirus, including one in which 500 mg/L of sulbactam was added. The last well was used as control of amoeba survival. After 24 h, amoebae were counted and Tupanvirus was titrated by qPCR as previously described^14^. In order to assess whether sulbactam could have affected newly formed virions, tupanviruses produced on amoebae incubated with sulbactam were inoculated on fresh amoebae at different concentrations. Their growth was monitored using high content screening microscopy every 8 h for 48 h^42^. Viral replication was compared to that of tupanviruses produced on amoebae non-treated with sulbactam at the same MOIs.

### Nuclease activity assessment

Nuclease activity was assessed using double-stranded DNA, (+) and (−) single-stranded DNAs, and single-stranded RNAs as substrates. Single-stranded DNAs were synthetic polynucleotides (see Supplementary Table 3); double-stranded DNA was obtained by annealing (+) and (−) single-stranded DNAs in a thermocycler at temperatures decreasing from 95 °C to 25 °C over 1 h. RNAs used as substrate were from *Escherichia coli*, from different bacteria that differ by the G + C content of their genomes (*Streptococcus parasanguinis* (41.8%), *Vibrio parahaemolyticus* (45.4%), *Vitreoscilla massiliensis* (49.4%), *Aeromonas salmonicida* (58.5%), *Aeromonas hydrophila* (61.5%) and *Pseudomonas aeruginosa* (66.6%)), and from *Acanthamoeba castellanii*. RNAs were purified using RNeasy columns (Invitrogen, Carlsbad, CA, USA). Enzymatic reactions were performed by incubating each polynucleotide (2 µg) with 15 µg of the expressed Tupanvirus protein TupBlac in Tris–HCl buffer 50 mM, pH 8.0, sodium chloride 0.3 M, using a final volume of 20 µL at 30 °C for 2 h. After incubation, the material was loaded onto denaturing polyacrylamide gel electrophoresis (dPAGE) at 12% or analysed using the Agilent RNA 6000 Pico LabChip kit on an Agilent 2100 Bioanalyzer (Agilent Technology, Palo Alto, CA, USA). Controls were carried out under the same conditions. The action of TupBlac on RNAs was also assayed in the presence of sulbactam (10 µg/mL), and of ceftriaxone (0.4 µM), an inhibitor of human metallo β-lactamase fold DNA repair nucleases SNM1A and SNM1B^23^, and of EDTA (10 mM). To do this, enzymatic reactions were conducted at 30 °C by incubating *E. coli* RNA (1 µg) with TupBlac (15 µg) in the presence of ceftriaxone at 200 µM. At different times, reactions were stopped by addition of proteinase K (10 µg) and incubated 1 h at 37 °C. Nuclease activity on various types of nucleic acids of an irrelevant recombinant protein, succinate dehydrogenase enzyme, produced and purified by the same process and collected in the same fractions as Tupanvirus beta-lactamase TupBlac and used as negative control was assessed. In addition, RNase activity of bacterial lysates purified by the same process and collected as Tupanvirus beta-lactamase and used as negative control was tested. For a quantitative assessment of the RNase activity of the TupBlac enzyme, we used the RNaseAlert QC System kit (Fisher Scientific, Illkirch, France) according to the manufacturer's protocol. This assay uses as substrate a fluorescence-quenched oligonucleotide probe that emits a fluorescent signal in the presence of RNase activity. RNase activities were assayed in the absence or presence of sulbactam (10 µg/mL) or ceftriaxone (200 µM). Negative controls were made with all the reagents used (RNase free water, enzyme buffer, sulbactam and ceftriaxone). Fluorescence was monitored continuously at 37 °C for 1 h by a Synergy HT plate reader (BioTek Instruments SAS, Colmar, France) with a 485/528 nm filter set. RNase activities of TupBlac were estimated using supplied RNase A used as a standard (10 mU/mL). Two independent experiments were conducted.

## Supplementary Information

Supplementary Information 1.

Supplementary Information 2.
