# Supplementary material for: A protein of the metallo-hydrolase/oxidoreductase superfamily with both beta-lactamase and ribonuclease activity is linked with translation in giant viruses
Source: Sci Rep. 2020 Dec 10;10:21685. doi: 10.1038/s41598-020-78658-8 (PMC7729979; doi:10.1038/s41598-020-78658-8)
Supplement: Supplementary file 1 — Supplementary Information 1. [file 41598_2020_78658_MOESM1_ESM.docx]

Supplementary Information TITLE PAGE

Full-length title: A protein of the metallo-hydrolase/oxidoreductase superfamily with both beta-lactamase and ribonuclease activity is linked with translation in giant viruses

Short title (for the running head): Metallo-beta-lactamase fold in giant viruses

**Author list: Philippe COLSON^1,2^, Lucile PINAULT^2^, Said AZZA^2^, Nicholas ARMSTRONG^2^, Eric CHABRIERE^1,2^, Bernard LA SCOLA^1,2^, Pierre PONTAROTTI^1,3^, Didier RAOULT^1,2^ ***

**Affiliations:** ^1^ Aix-Marseille Univ., Institut de Recherche pour le Développement (IRD), Assistance Publique - Hôpitaux de Marseille (AP-HM), MEPHI, 27 boulevard Jean Moulin, 13005 Marseille, France; ^2^ IHU Méditerranée Infection, 19-21 boulevard Jean Moulin, 13005 Marseille, France; ^3^ CNRS, Marseille, France

*** Corresponding author:** Prof. Didier Raoult, IHU - Méditerranée Infection, 19-21 boulevard Jean Moulin, 13005 Marseille, France. Tel.: +33 413 732 401, Fax: +33 413 732 402; email: didier.raoult@gmail.com

**This PDF file includes:**

Figures S1 to S8

Tables S1 to S3

File S1

SI References

**Fig. S1. Phylogeny reconstruction based on Tupanvirus deep ocean protein AUL78925.1, its homologs with the greatest BLASTp scores and various MBL superfamily members.**

Phylogeny reconstruction was performed after amino acid sequence alignment with the Muscle program [1] with the Maximum-Likelihood method using FastTree [2], and tree was visualized with the MEGA 6 software [3]. The amino acid sequences analyzed are Tupanvirus deep ocean protein AUL78925.1 and its homologs with the greatest BLASTp scores (see *SI Appendix*, Table S2), a set of previously described MBL fold proteins [4] and a set of sequences from the UniProtKB database [5] previously used for phylogeny reconstructions.

**Fig. S2. Amino acid alignment and structural prediction obtained using the Phyre2 web portal for protein modeling, prediction and analysis [6] for Tupanvirus deep ocean protein AUL78925.1.**

This figure was copied-pasted from the Phyre2 web portal (http://www.sbg.bio.ic.ac.uk/~phyre2/html/page.cgi?id=index)


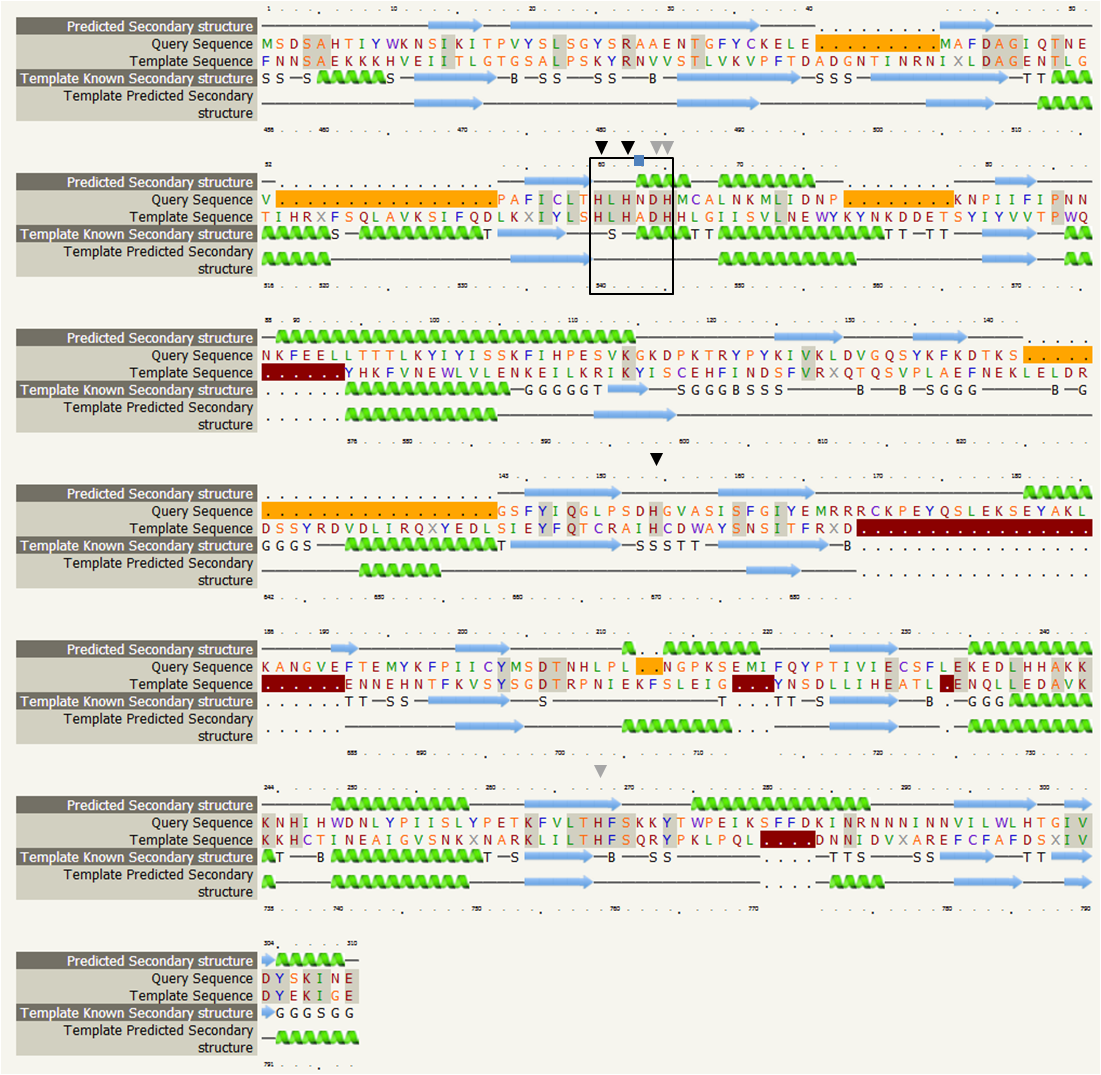


Aligned sequences are that of the recombinant protein AUL78925.1 of Tupanvirus deep ocean (named TupBlac) and that of the Phyre2 tool’s best match, a long form ribonuclease Z (RNase Z) from yeast (template c5mtzA). The conserved MBL motif "HxHxDH" in amino acid positions 60-65 is indicated by a black frame. Amino acids of the two putative metal-binding sites in positions H60-H62-H154 and D64-H65-H268 are indicated by black and grey point down triangles, respectively. A 3D modeling is available as Supplementary File S1 (3D_Tupanvirus_AUL78925_MBL_fold_protein.pdb).

**Fig. S3. Number of translation-associated components based on the absence or presence of an MBL fold protein.**

**
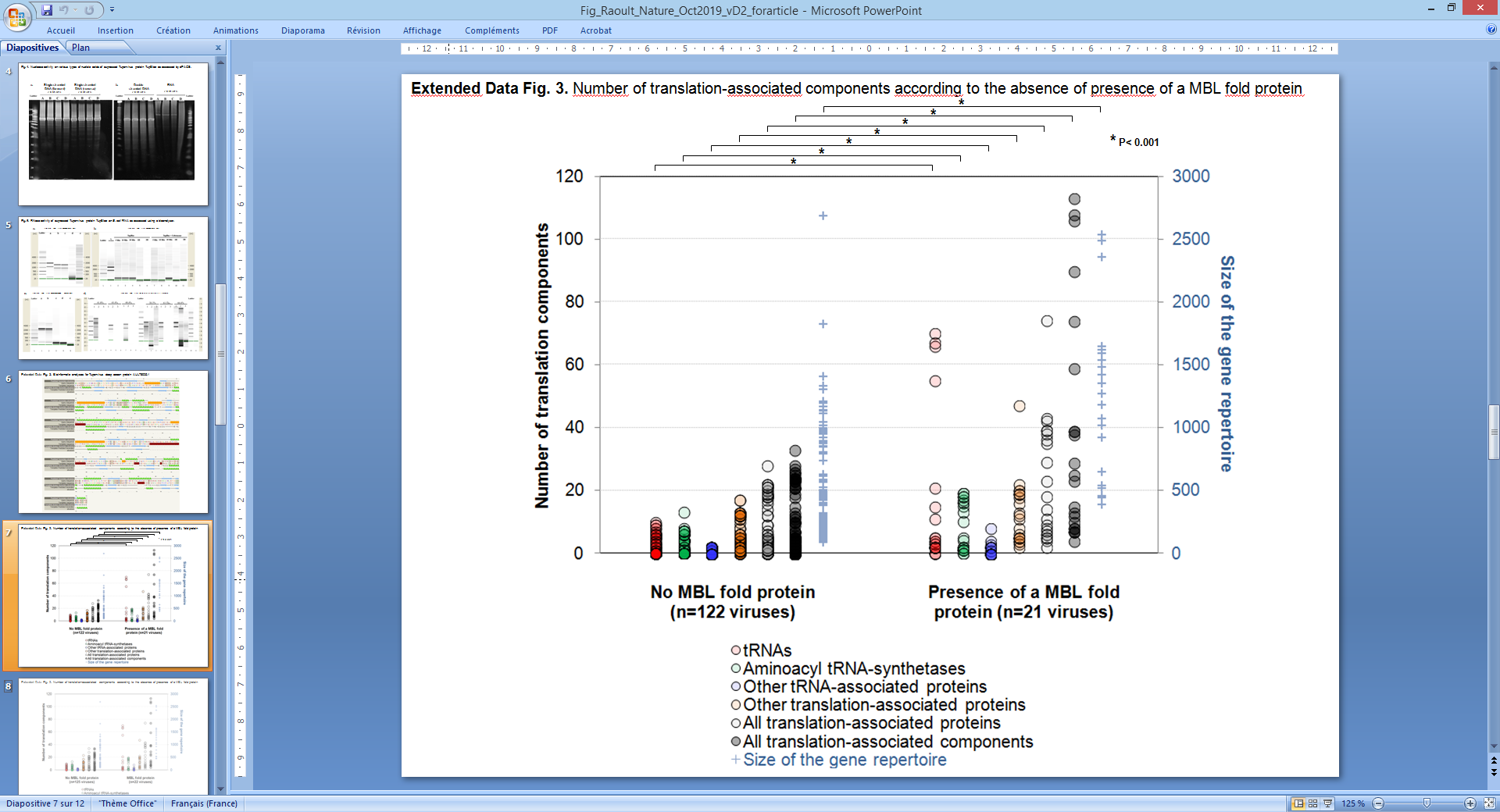
**

**Fig. S4. Hierarchical clustering based on the presence/absence of an MBL fold protein and other features of giant viruses (see also Extended Data Table 2).**

Hierarchical clustering was performed using the MultiExperiment Viewer software [7] based on the patterns of presence/absence of MBL fold protein, numbers of translation-associated components (number of tRNAs, aminoacyl tRNA-synthetases, other tRNA-associated proteins, other translation-associated proteins) and size of the gene repertoires for Megavirales members (SI Appendix, Table S2) (A). The MEGA 6 software [3] was used for visualization as a tree (B). Giant viruses with a MBL fold protein are indicated by a red bold font. For each item, the maximum value was determined, and values for each virus were considered relatively to these maximum values, being therefore comprised between 0 and 100%.

**A.**

**
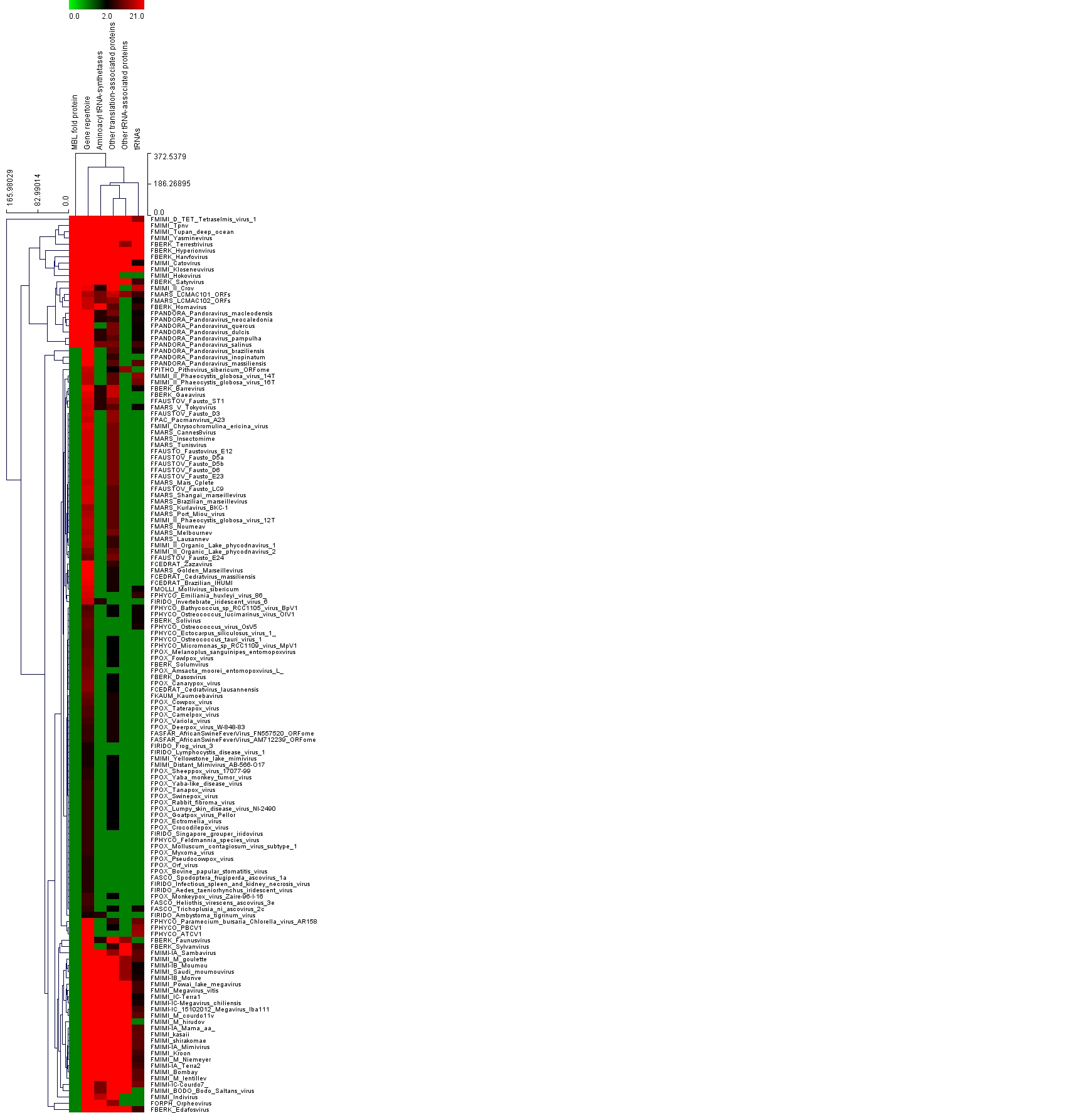
**

**B.**

**Fig. S5. Michaelis-Menten fitted data for determining kinetic parameters of TupBlac on the degradation of nitrocefin**

**Fig. S6. Tupanvirus deep ocean growth on *Acanthamoeba castellanii* strain Neff after a first passage on *A. castellanii* in the absence (a) or presence (b) of sulbactam.**

Viral growth was assessed by high content screening analysis as described in [8].

**
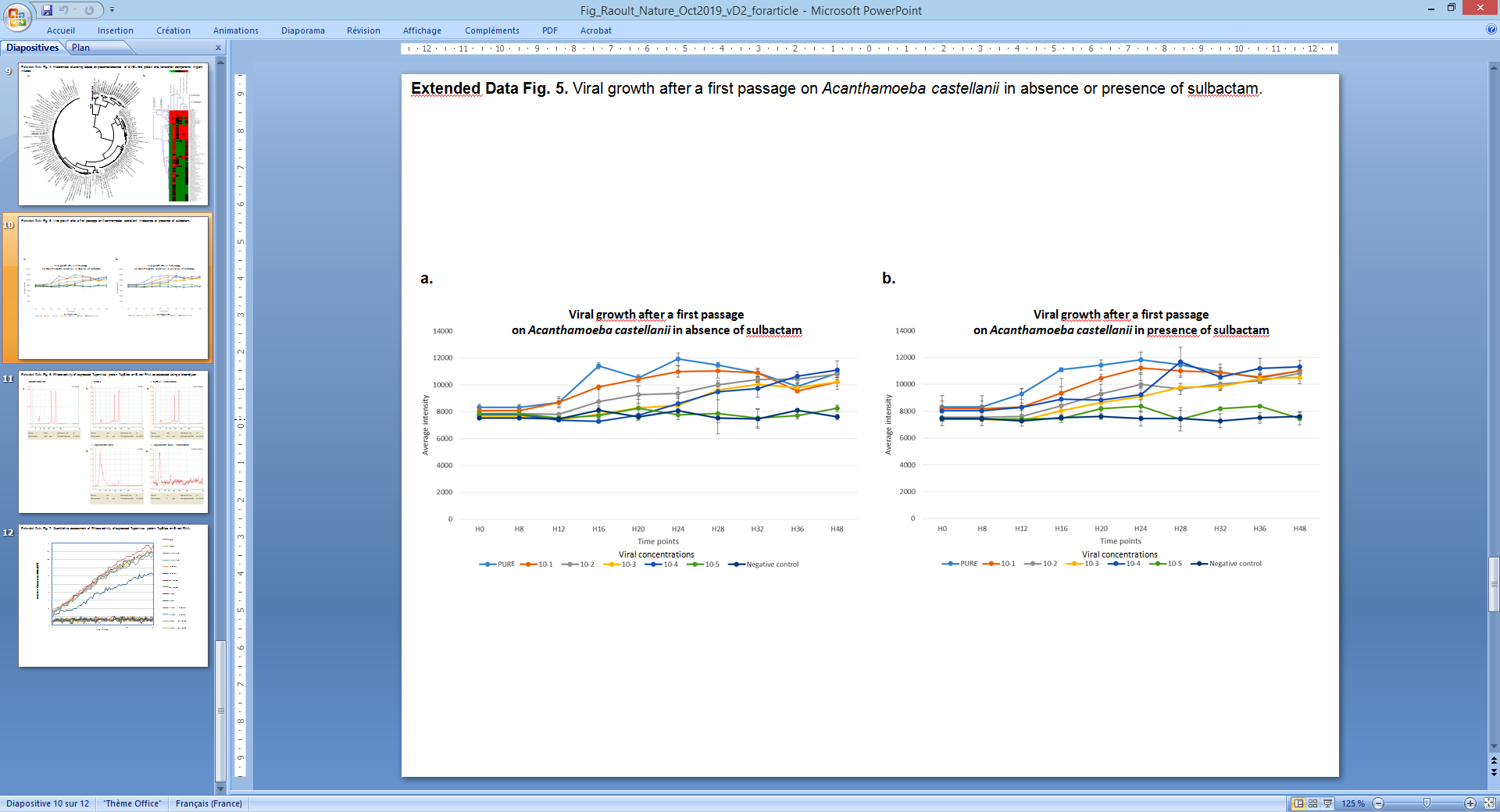
**

**Fig. S7. RNase activity of expressed Tupanvirus protein TupBlac on E. coli RNA, as visualized by a bioanalyzer.**

RNA samples (1 µg) incubated with 15 µg of TupBlac at 30°C in the absence or presence of 10 µg/mL of sulbactam or 200 µM of ceftriaxone. Nuclease activity was visualized as electrophoregrams performed using the Agilent Bioanalyzer 2100 with the RNA 6000 Pico LabChip (Agilent Technologies, Palo Alto, CA). a: no treatment (a); buffer (b); sulbactam (c); TupBlac in the absence (d) or presence (e) of sulbactam. See also Figure 4a for representation from the same data as digital gel image.

**
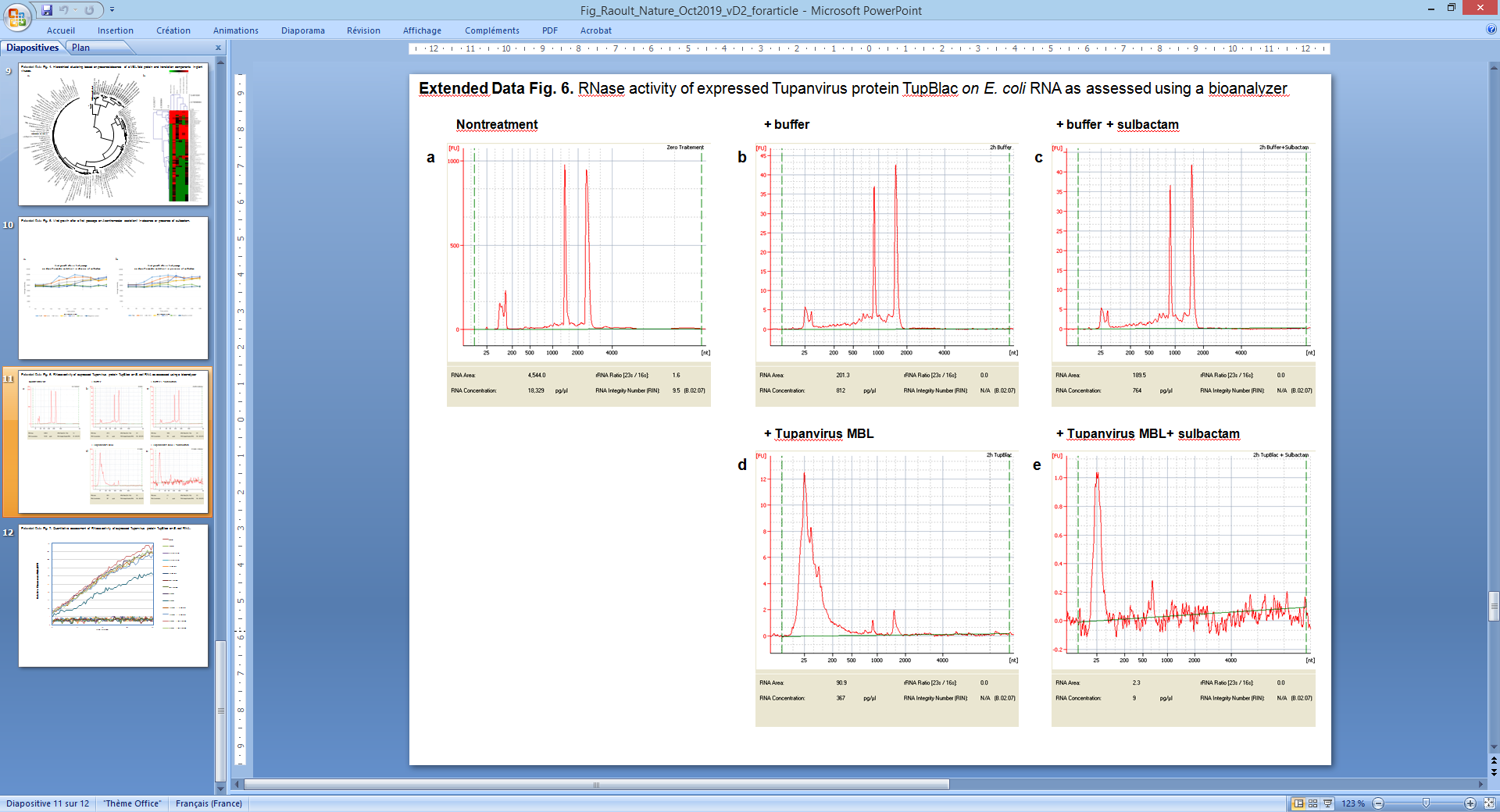
**

**Fig. S8. Quantitative assessment of RNase activity of expressed Tupanvirus protein TupBlac on E. coli RNA.**

The RNase activity of TupBlac enzyme was measured using the RNaseAlert QC System kit (Fisher Scientific, Illkirch, France) according to the manufacturer's protocol. Fluorescence was monitored continuously at 37°C for 1h in Synergy HT plate reader (BioTek Instruments SAS, Colmar, France) with a 485/528 nm filter set. Two independent experiments were conducted. The addition of TupBlac was associated with a significant increase in fluorescence compared to all controls used (RNase-free water, enzyme buffer, sulbactam, and ceftriaxone). No inhibition of RNase activity of TupBlac was detected with sulbactam or ceftriaxone.

**
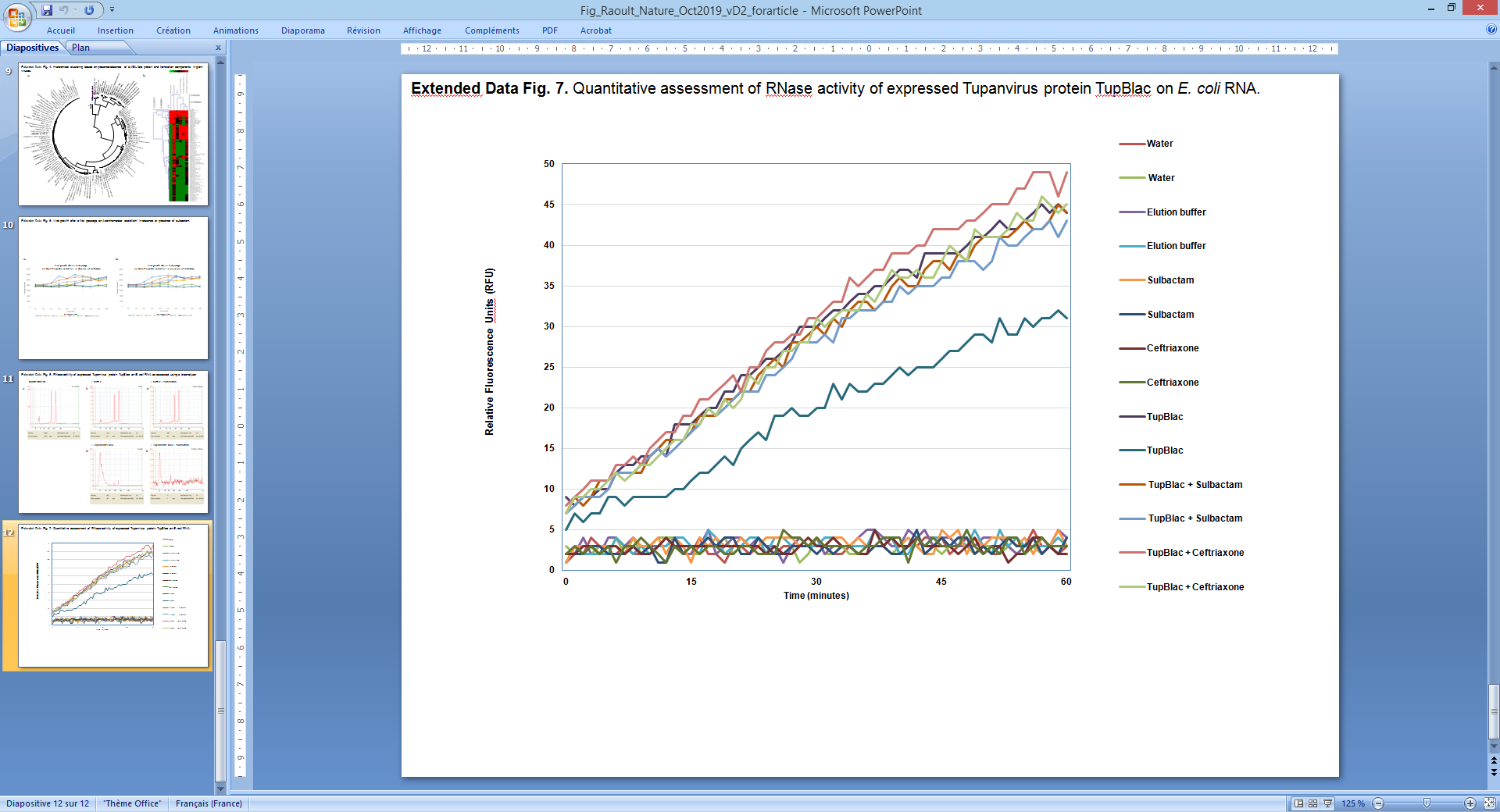
**

**Table S1. Homologs with the 100 greatest BLASTp scores for Tupanvirus deep ocean protein AUL78925.1.**

*Supplementary* *Table S1 - continued*

**Table S2. Presence or absence of a MBL fold protein, size of the gene repertoire, and number of translation-associated components among Megavirales members.**

*Supplementary* *Table S2 - continued*

*Supplementary* *Table S2 - continued*

**Table S3. Sequence of synthetic DNA (130 nucleotides) used in enzyme treatments as substrates as a single strand or double strand obtained by annealing forward with reverse DNA synthetic.**

| Name | Sequence |
| --- | --- |
| DNA_Synt_Fwd | ATAGAACAACCAAAAAAATATCAAAATCTAGAGATGAATCAAGTGAATCAGAAGAATCTGATAATGAATCTGATAATGAATCCGATGAGGAAGTTGAATCAGAAACTGAGATAGAACCAGTCAAATCTAA |
| DNA_Synt_Rev | TTAGATTTGACTGGTTCTATCTCAGTTTCTGATTCAACTTCCTCATCGGATTCATTATCAGATTCATTATCAGATTCTTCTGATTCACTTGATTCATCTCTAGATTTTGATATTTTTTTGGTTGTTCTAT |

**File S1. Tridimensional modeling for Tupanvirus deep ocean protein AUL78925.1.**

This file (named “3D_Tupanvirus_AUL78925_MBL_fold_protein.pdb”) was obtained from the Phyre2 web portal (http://www.sbg.bio.ic.ac.uk/~phyre2/html/page.cgi?id=index)

**References**

1. Edgar RC. MUSCLE: a multiple sequence alignment method with reduced time and space complexity. *BMC Bioinformatics* 2004; **5**: 113.

2. Price MN, Dehal PS, Arkin AP. FastTree 2--approximately maximum-likelihood trees for large alignments. *PLoS One* 2010; **5**: e9490.

3. Tamura K, Stecher G, Peterson D *et al.* MEGA6: Molecular Evolutionary Genetics Analysis version 6.0. *Mol Biol Evol* 2013; **30**: 2725-2729.

4. Diene SM *et al.* Human metallo-ß-lactamase enzymes degrade penicillin. *Sci Rep* 2019; **9**: 1273.

5. Alderson RG, Barker D, Mitchell JB. One origin for metallo-beta-lactamase activity, or two? An investigation assessing a diverse set of reconstructed ancestral sequences based on a sample of phylogenetic trees. *J Mol Evol* 2014; **79**: 117-129.

6. Kelley LA, Mezulis S, Yates CM *et al.* The Phyre2 web portal for protein modeling, prediction and analysis. *Nat Protoc* 2015; **10**: 845-858.

7. Saeed AI, Sharov V, White J *et al.* TM4: a free, open-source system for microarray data management and analysis. *Biotechniques* 2003; **34**: 374-378.

8. Francis R, Ominami Y, Bou Khalil JY *et al.* High-throughput isolation of giant viruses using high-content screening. *Commun Biol* 2019; **2** 216-0475.
